# Cardiovascular genetic counselor decision making about discussing life insurance with patients

**DOI:** 10.1002/jgc4.70146

**Published:** 2025-11-25

**Authors:** Sara Cherny, Sarah Jurgensmeyer Langas, Miguel Moran, Susan Christian, Gregory Webster

**Affiliations:** ^1^ Division of Cardiology Ann & Robert H. Lurie Children's Hospital of Chicago Chicago Illinois USA; ^2^ Department of Pediatrics, Feinberg School of Medicine Northwestern University Chicago Illinois USA; ^3^ Department of Medical Genetics University of Alberta Edmonton Alberta Canada

**Keywords:** cardiovascular genetics, genetic counseling, insurance discrimination, life insurance

## Abstract

Genetic counselors (GCs) educate patients about the benefits, risks, and limitations of genetic testing. The regulatory environment governing the use of genetic data in life insurance is not uniform internationally or within the United States (US). This multinational survey assessed how cardiovascular GCs incorporate the topic of life insurance (LI) into patient discussions. An online survey was distributed to GCs currently providing care to patients with non‐syndromic cardiovascular disease. Brief clinical scenarios were included to avoid participants considering ambiguous or marginal phenotypes. Respondents were 121 cardiovascular GCs from five countries. Patient phenotype was the strongest indicator of whether GCs engaged in LI discussion. For phenotype‐negative pediatric and adult patient scenarios, 62% and 74% of participants would discuss LI. For phenotype‐positive pediatric and adult patient scenarios, 29% and 39% of participants would discuss LI. Non‐U.S. participants were more likely to discuss LI with phenotype‐positive patients than U.S. participants (61% vs. 33%, *p* = 0.005). Participants seeing primarily adult patients were more likely to discuss LI than those seeing primarily pediatric patients, for both pediatric (44% vs. 12%, *p* = 0.003) and adult phenotype‐positive scenarios (46% vs. 17%, *p* = 0.008). Most participants would discuss LI with family variant testing (91%). Many participants reported patients declining genetic testing due to fear of genetic discrimination (77%) and 21% reported patients who were denied LI due to a genetic test result. Insufficient time was an important reported reason to not discuss LI (31%). Most participants reported learning about LI considerations in graduate education and reported confidence in their knowledge and ability to learn about related laws. Patient phenotype was the primary driver of whether cardiovascular GCs discussed life insurance implications of genetic testing with their patients, regardless of the age of the patient or the nationality of the genetic counselor. This study is the first to assess this nuanced aspect of cardiovascular genetic counseling and may support GC practice decisions and education.

**What is known about this topic?**

Patients and genetic counselors consider genetic discrimination in decision making. Genetic counseling training programs include education about insurance discrimination.

**What this paper adds to the topic?**

This paper adds quantitative data about the practices of cardiovascular genetic counselors regarding the discussion of life insurance with patients. It reports the relevance of patient phenotype, age of patient, setting of provider, and geographical location of provider.

## 1 INTRODUCTION

The importance of protecting individuals from genetic discrimination, a unique form of discrimination based solely on apparent or perceived genetic variation, is demonstrated by decades of ethical and medical research (Billings et al., 1992, Natowicz et al., 1992). The Genetics Information Non‐discrimination Act (GINA) of 2008 in the United States (US) and others in numerous countries including Canada, Belgium, Norway, Australia, Denmark, France, Lithuania, Portugal, Sweden, the United Kingdom (UK), and the Netherlands aim to prevent genetic discrimination. The Dutch Medical Examination Act demonstrated early awareness of the potential use of genetic information by insurers, and restricted the use of genetic test results in life insurance determinations (Geelen et al., 2012). Canada, the United Kingdom, and Australia do not currently permit the use of genetic test information for life insurance underwriting; however, these are temporary injunctions and continue to be evaluated and studied (Mittra 2006; Dowling et al., 2022; Tiller et al., 2021; Cowan et al., 2022; Jones, 2024). Although GINA in the United States focuses on health insurance and excludes life insurance protection, several U.S. states have enacted or are currently evaluating bills to prevent discrimination in life insurance, a financial product held by 51% of Americans in 2024 (Anderson 2021; Genetic Discrimination Observatory, n.d.; LIMRA, 2024).

Despite these laws, consumers continue to report fear of genetic discrimination. Allain et al. (2012) showed that fear of insurance discrimination was the second most common reason for declining genetic testing in a cancer cohort. Lenartz et al. (2021) surveyed the U.S. general population in 2020 and found that understanding of GINA was low while concern for genetic discrimination was persistently reported. Recently, Muller et al. (2024) concluded that the 2019 Australian partial moratorium on the use of genetic information in life insurance was insufficient to allay fears about genetic discrimination or adequate access to life insurance products. This is consistent with the findings by Tiller and colleagues that Australian consumers overwhelmingly believe life insurers should not be allowed to use genetic results in life insurance underwriting and that federal legislation is required to regulate this area (Tiller et al., 2024). Further work assessing the impact of these laws is ongoing in Canada. Fernando et al. (2024) found that the Genetic Non‐Discrimination Act (GNDA) changed some life insurer behavior (e.g., new policy definitions of genetic testing, consent language, and result disclosure requirements). However, they note that insurers may continue to use orthogonal methods such as family history questions and broad language that circumvent the law or confuse applicants.

The issue of life insurance is relevant to cardiovascular genetics patients due to the life‐shortening risks of heritable cardiac diseases, the value of specific therapeutic interventions in preventing morbidity and mortality, and the emphasis on cascade testing for individuals without cardiac symptoms at the time of testing. As documented in a scoping review by Joly et al. (2013), studies conducted as early as 1991 considered the relationship between patient insurability and heritable cardiac diseases such as familial hypercholesterolemia, Marfan syndrome, and hypertrophic cardiomyopathy. Joly noted that studies are often gene or condition‐specific and highlight the difficulty in generalizing study findings when genetic conditions vary in penetrance, age of onset, and severity. More recently, Mohammed et al. (2017) assessed patients with, or at risk for, sudden arrhythmia syndromes and found that approximately a third of their cohort were denied life insurance, with most reporting the cause of denial being a pre‐existing condition. Important context for insurance considerations in heritable cardiac disease is that these conditions exhibit highly variable penetrance within and between families. Wauters et al. highlight this in their 2016 review, noting that while patients and families appear to not distinguish between “symptomatic” and “asymptomatic” individuals, legislation does. (Wauters et al. 2016).

There is currently limited research on how genetic counselors discuss genetic discrimination with their patients. Pfeffer et al. (2003) reported that 24 of 25 cancer genetic counselors in their study reported that they either always (64%) or almost always (32%) discussed genetic discrimination with their patients. However, 8 years later, 3 years after the passing of GINA in the United States, Pamarti et al. (2011) surveyed a diverse group of genetic counselors and found that less than half of the 257 genetic counselors reported incorporating GINA and genetic discrimination in their counseling. These authors reported that cancer counselors were covering this information most often (Pamarti et al., 2011). While these studies addressed genetic discrimination laws in general, within the genetic counseling community there is insufficient information available to identify the standard of care for discussing life insurance. We surveyed cardiovascular genetic counselors to determine their current practices regarding discussion of life insurance implications of genetic testing with their patients.

## 2 METHODS

### 2.1 Participants and recruitment

A REDCap web‐based, English‐language survey was distributed to members of the National Society of Genetic Counselors (NSGC) via email listserv on June 12, 2024. Additional distribution methods included posting the survey on social media accounts of the research team and snowball sampling via email to reach additional genetic counselors who treat cardiovascular patients. The survey was open for 1 month (6/12/24–7/12/24) and one pre‐specified reminder email was sent after 2 weeks.

Genetic counselors who reported caring for patients with or at risk for non‐syndromic heritable cardiac disease, identified as arrhythmia, cardiomyopathy, aortopathy, and familial hypercholesterolemia were recruited. Genetic counselors providing genetic counseling exclusively for patients with syndromic causes of heart disease such as 22q11 deletion syndrome were excluded. Participants were asked whether they predominantly saw pediatric (0–17 years) or adult patients (18+ years) and these self‐reported identifiers were used for analysis.

The study was submitted for human subjects evaluation at Lurie Children's Hospital (#2024‐6785) and was determined to be exempt from IRB review.

### 2.2 Survey design

Previously published survey measures were evaluated for key questions that could be adapted for this purpose (Mohammed et al., 2017; Tiller et al., 2024). After initial development, the survey was piloted with four non‐cardiology genetic counselors and feedback from pilot surveys was incorporated into the final survey (Table S1). Non‐cardiology genetic counselors were sought for the pilot in order not to limit the final potential sample size.

The 20‐question survey was divided into four sections: clinical practice demographics, clinical practice and life insurance, training and resources regarding life insurance, and participant demographics. Questions included multiple choice, Likert scale, and yes/no questions. Likert scale answers were {Always, Often, Sometimes, Rarely, Never, N/A} and {Very much agree, Somewhat agree, Neither agree nor disagree, Somewhat disagree, Disagree}. Participants were asked to respond to questions about both pediatric and adult patients. While no specific questions were required for completion of the survey, participants that did not complete at least 75% of the survey (up to question 17) were excluded. Brief clinical scenarios based on long QT syndrome and a pathogenic variant in *KCNQ1* were provided for each example of “phenotype‐positive” or “phenotype‐negative” contexts to assess the impact of phenotype on decision making. Scenarios were designed to avoid ambiguous or marginal phenotypes.

### 2.3 Data analysis

Statistical analyses were performed using IBM SPSS Statistics, Version 29, and Microsoft Excel. Descriptive statistics were used to summarize demographics and genetic counselor practices. Likert scales were dichotomized into groups as follows: Always/Often and Sometimes/Rarely/Never, and Very much agree/Somewhat agree and Neither agree nor disagree/ Somewhat disagree/Disagree (Table S2). We conducted a sensitivity analysis for our primary findings to ensure that our Likert dichotomization did not introduce inadvertent bias (Table S3). Chi‐squared analyses determined statistically significant differences between United States and non‐United States as well as adult and pediatric genetic counselors' practices. A *p*‐value <0.05 was considered statistically significant.

## 3 RESULTS

### 3.1 Demographics

The survey was accessed by 152 individuals and responses from 121 participants were retained for analysis (79%). We used published data from the NSGC to approximate the total number of genetic counselors in cardiology who would have been eligible to respond, based on self‐report of cardiology as a primary area of practice (*N* = 349) (National Society of Genetic Counselors, 2024). Therefore, our 121 participants represent a response rate of approximately 35%. Three‐quarters of participants were from the United States (*n* = 89; 74%, Table S4) and a quarter of participants (*n* = 32) were from other countries including Canada (*n* = 17, 14%), the United Kingdom (*n* = 9, 7%), Australia (*n* = 5, 4%), and New Zealand (*n* = 1, 1%), Table 1. The median year of graduation from genetic counselor training was 2016 (range: 1982–2023) which is similar to the reported median in the 2024 NSGC Professional Status Survey. Participants primarily worked in academic centers (*n* = 82, 68%) and provided care to adult patients (*n* = 95, 79%).

**TABLE 1:** Participant Demographics.

	*N* (%)
Country	
Domestic	89 (74)
United States	89 (74)
International	32 (26)
Canada	17 (14)
United Kingdom	9 (7)
Australia	5 (4)
New Zealand	1 (1)
Practice setting	
Academic Center	82 (68)
Non‐academic Center	23 (19)
Other	15 (13)
Patient age group predominantly seen	
Adult	95 (79)
Pediatric	26 (21)
Types of non‐syndromic patients seen*	
Cardiomyopathy	118 (98)
Arrhythmia	113 (93)
Aortopathy/connective tissue disorder	96 (79)
Familial hypercholesterolemia	87 (72)
Congenital heart disease	64 (53)

*Note*: Results sum to >100% when multiple answers were permitted (*).

### 3.2 The importance of phenotype

**FIGURE 1:**
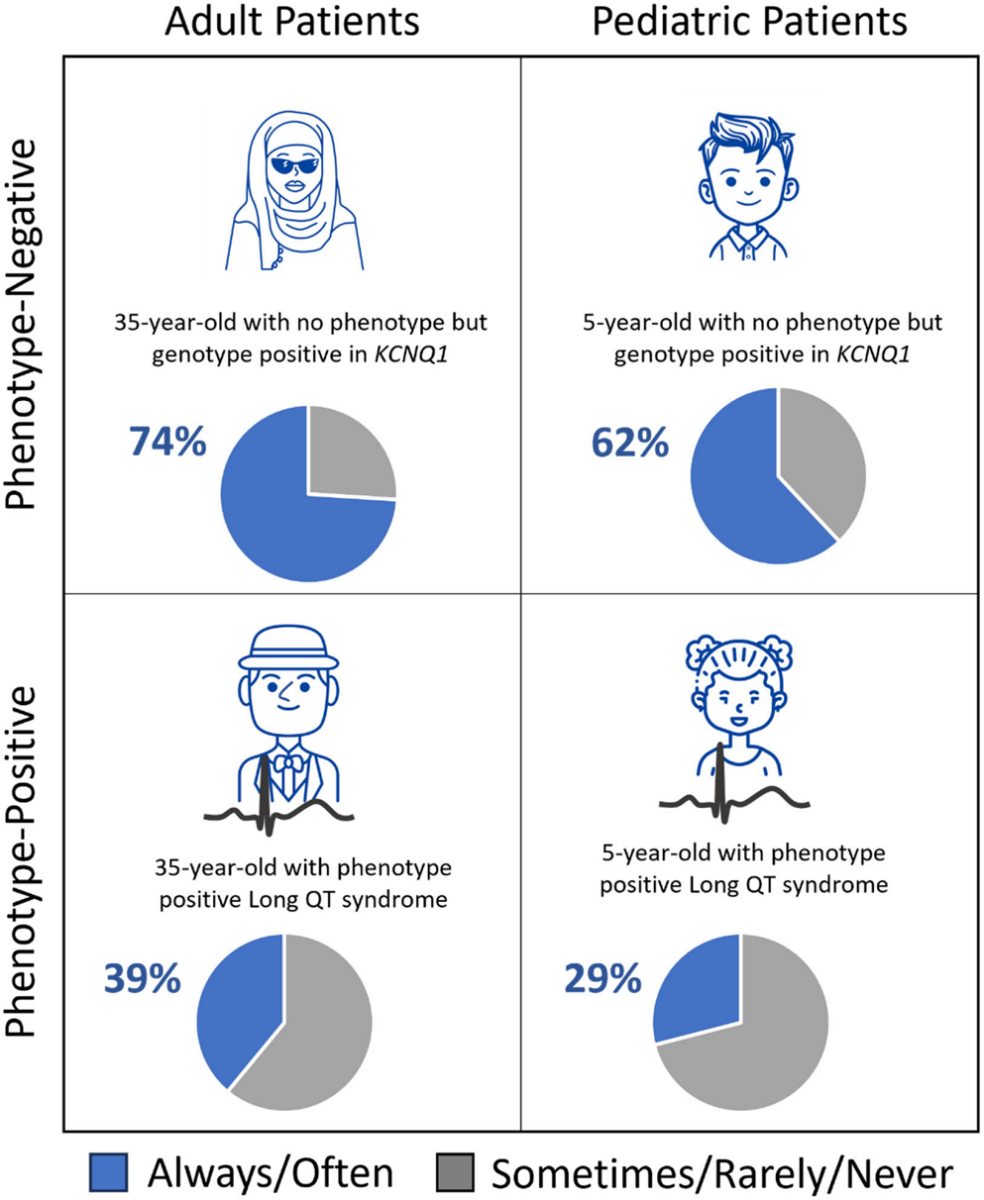
Phenotype‐negative patients were more likely to trigger conversations about life insurance.

Over 90% of participants reported that phenotype always/often contributed to whether they discussed life insurance with their patients (*n* = 109, 91%). Brief clinical scenarios where patients were offered genetic testing but did not personally have a clinical phenotype (phenotype‐negative scenarios) were most likely to elicit a response that genetic counselors always/often discussed life insurance. For pediatric phenotype‐negative scenarios, 75/121 participants reported that they would always/often discuss life insurance with patients (62%) and in phenotype‐negative scenarios with adult patients, 89/121 reported that they would always or often discuss life insurance (74%), Figure 1. Fewer participants reported that they always/often would discuss life insurance if the patient were phenotype‐positive (pediatric scenarios *n* = 35, 29%; adult scenarios *n* = 47, 39%).

Most participants reported always/often discussing life insurance in the setting of family variant testing (*n* = 109, 91%). However, about half of participants reported always/often discussing life insurance when ordering a panel (*n* = 64, 54%) or exome/genome sequencing (*n* = 65, 55%). For panel testing specifically, participants seeing predominantly adult patients were more likely to report always/often discussing life insurance compared to participants seeing predominantly pediatric patients (adult genetic counselors *n* = 56, 61%; pediatric genetic counselors *n* = 8, 31%; *p* = 0.005).

Responses about phenotype were stratified by whether the participant predominantly saw adult or pediatric patients (Table 2). With regard to phenotype‐positive patients, regardless of patient age, participants who predominantly saw adult patients were more likely to report that they always/often discuss life insurance as compared to participants who predominantly saw pediatric patients. No difference was observed between these groups for phenotype‐negative patients.

**TABLE 2:** Impact of patient phenotype on survey responses, stratified by participant primary clinical concentration.

Patient age and phenotype	*N*	Responses, “Always” or “Often”		*p*‐Value
		GCs, Adult concentration	GCs, Pediatric concentration	
Adult scenario, phenotype‐positive	117	43 (46%)	4 (17%)	0.01*
Pediatric scenario, phenotype‐positive	98	32 (44%)	3 (12%)	0.003*
Adult scenario, phenotype‐negative	114	71 (79%)	18 (75%)	0.68
Pediatric scenario, phenotype‐negative	98	56 (78%)	19 (73%)	0.63

*Note*: “Adult/Pediatric Scenario” refers to the age of the patient in the clinical scenario provided in the survey. “Adult/Pediatric Concentration” refers to the self‐reported practice concentration for survey participants. Likert scale responses {Always, often, sometimes, rarely, never} were dichotomized as always/often versus other responses.

* Significant at *p* < 0.05.

Responses were also stratified by country of practice as shown in Table 3. Non‐U.S. participants were more likely to always/often discuss life insurance compared to U.S. participants for phenotype‐positive adult patients. Non‐U.S. and U.S. participants reported discussing insurance with the remaining patient types described in the clinical scenarios at a similar rate.

**TABLE 3:** Impact of patient phenotype on survey responses, stratified by participant geographic location.

Patient age and phenotype	*N*	Responses “always” or “often”		*p*‐Value
		GCs, Domestic	GCs, International	
Adult scenario, phenotype‐positive	117	28 (33%)	19 (61%)	0.005*
Pediatric scenario, phenotype‐positive	98	23 (32%)	12 (46%)	0.20
Adult scenario, phenotype‐negative	114	64 (77%)	25 (81%)	0.69
Pediatric scenario, phenotype‐negative	98	56 (79%)	19 (70%)	0.37

*Note*: “Adult/Pediatric Scenario” refers to the age of the patient in the clinical scenario provided in the survey. Likert scale responses {Always, often, sometimes, rarely, never} were dichotomized as always/often versus other responses.

* Significant at *p* < 0.05.

Participants were asked to report other factors related to whether life insurance was discussed with patients. After phenotype, the second and third most reported factors were forgetting (*n* = 60, 50%) and not having enough time (*n* = 38, 31%).

### 3.3 Patient concerns about and experience with life insurance

Three‐quarters of participants (*n* = 93, 77%) reported having a patient who declined genetic testing due to concerns about life insurance eligibility. U.S. participants (*n* = 73, 83%) were more likely to report a patient who declined genetic testing than non‐U.S. participants (*n* = 20, 63%, *p* = 0.018). In addition, 21% (*n* = 25) of participants reported having at least one patient who was denied life insurance because of a genetic test result. In this case, responses were similar for U.S. participants (*n* = 19, 21%) and non‐US participants (*n* = 6, 19%).

### 3.4 Resources, training, and institutional policies

Regardless of country of practice, most participants reported that they somewhat/very much agreed that they knew where to identify information about genetic non‐discrimination laws in their country (*n* = 106, 88%). For U.S. participants, when it came to identifying information at the state level, fewer than two‐thirds responded that they somewhat/very much agreed that they knew where to do so (*n* = 52, 59%).

Most participants reported that they had training in their graduate education about non‐discrimination laws related to genetic testing results (*n* = 94, 78%) and most reported having training in their graduate education about life insurance discrimination (*n* = 96, 79%). Of the participants who reported they had not received education in graduate school about genetic or insurance discrimination, all but one graduated prior to the enactment of GINA in 2008 (*n* = 9).

Participant responses were mixed regarding which entities should be responsible for providing education and information about genetics and life insurance. Many participants selected professional societies (42%) and genetic counseling graduate programs (31%). All options were selected by at least one participant, including genetic counseling regulation boards, employing institutions, department leadership, and life insurance companies.

A minority of participants (*n* = 16, 12%) reported an institutional or departmental policy regarding communication with patients about the life insurance implications of genetic testing.

## 4 DISCUSSION

In our study, patient phenotype stood out as the most important factor in cardiovascular genetic counselor decisions about whether to discuss life insurance with patients; it was more important than patient age and more important than the practitioner's geographic location. The primacy of phenotype‐negative status in our survey is consistent with prior studies of patient experiences (Christiaans et al., 2010; Mohammed et al., 2017; Pfeffer et al., 2003); however, these earlier studies either address a narrow group of phenotypes or focus on patient experience. Our results expand on the previous literature by surveying a large group of genetic counselors in a previously unassessed subspecialty, cardiovascular genetics, and exploring the impact of other factors including patient age, test type, and provider setting. This study contributes novel insights about the intersection of genetic counseling practice and life insurance.

It has been demonstrated in previous literature (Christiaans et al., 2010; Mohammed et al., 2017; Pfeffer et al., 2003) that patients who have a clinical cardiac diagnosis are at risk of being denied life insurance. An abnormal ECG, a clinical diagnosis of a cardiomyopathy, or a history of cardiac arrest may be sufficient for a life insurance provider to change their assessment of an applicant, regardless of the underlying etiology for those phenotypes. Our data reinforces this, with genetic counselors less frequently indicating that they would discuss life insurance with phenotype‐positive than phenotype‐negative patients in the clinical scenarios.

The importance of phenotype, or the lack of one, is also supported by our finding that over 90% of our participants discuss life insurance in the setting of family variant testing. Cascade genetic testing for a known family variant is unique in that it is often conducted on individuals with no apparent disease, and a positive result does not predict if or when an individual may develop a phenotype. Genotype‐positive, phenotype‐negative patients are, in theory, protected by the policies limiting the use of genetic testing in life insurance emerging in US states and in countries such as Australia. However, as noted in Tiller et al. (2024), even after the Australian moratorium, fears of genetic discrimination persisted. Further, the work from Geelen et al. (2012) showed that individuals and families see genetic information in context with not only phenotype, but also family history and social context. Another important topic raised by previous researchers is the idea that genetic information is not simply one's diagnosis or genetic test results. The impact of family history of cardiac arrhythmia syndromes on life insurance policies was assessed by Mohammed et al. (2017), and findings demonstrated that 50% of interviewees reported life insurance discrimination for unaffected family members. A recent study from researchers in Canada showed that risk assessments from breast cancer risk calculators, which include the presence of a pathogenic variant in their calculations, may be used to discriminate against individuals who wish to obtain life insurance (Reveiz, 2025). (Prince and Berkman, 2012) addressed the challenges of legally and medically defining manifest disease, highlighting the complexity inherent in attempting to define conditions that vary in symptomatology and age of onset, with many gene‐positive individuals remaining disease‐free their entire lives. (Prince and Berkman, 2012) Legislative efforts that narrowly define genetic information (e.g. GNDA addresses genetic test results only) may be insufficient to address the complex interaction of genotype with other factors.

At this time, there is no published data regarding how or to what extent the presence of a positive genetic test result, with or without a concurrent clinical phenotype, changes the calculations that are involved in life insurance determination. The genotype itself is relevant in at least some insurance decisions, highlighted by our finding that about 20% of our participants reported a patient denied coverage due to a genetic test result. However, since a pathogenic variant does not guarantee clinical disease due to reduced penetrance commonly seen in heritable cardiac diseases, the presence of one should not force unaffected patients out of a low‐risk insurance pool. Until insurers can understand the nuances of cardiogenetic phenotypes, genotypes, and penetrance, we agree with previous authors that changing insurability based exclusively on genetic data is a critical mistake (Prince et al., 2017; Rothstein, 2018).

An initial goal of the study was to assess whether there are meaningful differences in the practices of cardiovascular genetic counselors who see primarily pediatric patients as compared to those who see primarily adult patients. Since a pediatric patient is a non‐consenting minor, conversations about life insurance would occur with the parent and not the patient themselves, inherently changing the interaction. In addition, comprehensive genetic tests such as exome and genome sequencing are more likely to be ordered in the pediatric setting. In our survey, participants who primarily identified as caring for adult patients were more likely to prioritize discussions of life insurance in the clinical scenarios. And yet, as discussed recently by Ormond et al. (2023), children are a vulnerable population and caring for children with genetic conditions involves ethical considerations beyond genetic testing decision making itself. Our results suggest a core responsibility for those providing pediatric care. One's genetic code is currently a fixed, biological feature; therefore, decisions made in childhood can have lasting implications. An important task in supporting lifelong autonomy for a child, a priority for pediatric care, is to include thoughtful considerations of all potential risks and benefits of genetic testing (Committee on Bioethics et al., 2013). Thus, we suggest that pediatric genetic counseling conversations, especially in phenotype‐negative patients, should incorporate life insurance discussions.

Providing the right information to support patient autonomy in the context of genetic testing is a core component of genetic counseling. Genetic counselors, like most medical providers, must weigh the value of many important messages that they wish to impart to their patients within the time available to do so. Accordingly, our participants reported that forgetting to discuss life insurance and time available to do so were major limiting factors in whether or not discussion about life insurance was included in their sessions. While our data indicate that practicing genetic counselors should prioritize clinic time for life insurance discussions, particularly for phenotype‐negative patients, more data will be needed to identify specific barriers and differences such as between providers in adult versus pediatric settings.

It is unsurprising that three‐fourths of our participants reported that one or more patients declined genetic testing due to fear of discrimination. A number of other authors from multiple countries have documented patient fear of genetic discrimination, particularly in insurance access, over the past decade (Allain et al., 2012; Geelen et al., 2012; Joly et al., 2013; Muller et al., 2024; Prince et al., 2021). More United States‐based practitioners reported patient(s) who declined genetic testing due to fear of life insurance discrimination (83% vs. 63%). While this study does not provide data on why these differences exist, it may be due to the presence of protective national laws in countries such as the United Kingdom, Canada, and Australia. Over time we hope to better understand the utility and effectiveness of these legislative actions.

Our final area of inquiry was regarding genetic counselor training and education about life insurance discrimination. It is reassuring that genetic counselors in this survey reported that current training programs are providing adequate support, with 78% endorsing the content of their own training programs and 88% knowing where to find more information about non‐discrimination laws. That said, U.S. genetic counselors were less likely to endorse that they knew where to find material about insurance discrimination at the state level, indicating a need for easier access to this information. As our participants indicated, there are a number of organizations for genetic counselors in the United States and other countries that could take responsibility to increase awareness and education in this area.

At this time, there are no professional guidelines to inform genetic counselor practice regarding life insurance discussions. The Accreditation Council for Genetic Counseling (ACGC) in North America requires that graduates are competent in understanding inequities of healthcare systems as well as financial considerations in the delivery of genetic services (ACGC, 2023) which could be extrapolated to include insurance considerations. In 2020, the National Society of Genetic Counselors published a position statement that “recognizes the use of genetic information in insurance underwriting for life, long‐term care, and disability insurance plans, but advocates for transparency in the underwriting process, and believes that the use of genetic information must be based on strong actuarial evidence for disease risk.” (NSGC, 2020) Further, the American College of Medical Genetics and Genomics (ACMG) published a statement in 2022 that addresses the use of genetic and family history information in life insurance underwriting (Seaver et al., 2022). These documents indicate an important direction from genetic counseling leadership and governance. Our data adds to the evidence that more guidance and education in this area are needed. Further, genetic counselors may not be the only medical providers engaging in these discussions. Information regarding national and state‐level protections against insurance discrimination should be accessible for any clinician.

### 4.1 Limitations

The participant pool was inherently limited by the number of cardiovascular genetic counselors providing direct patient care, limiting the generalizability. Recruitment may have also been affected by selection bias, as genetic counselors more interested in or experienced with life insurance conversations may have been more likely to participate. We provided phenotype‐positive and phenotype‐negative clinical scenarios, but clinical practice is often less dichotomous and requires careful counseling and expert team collaboration. Future efforts are likely to require a more standardized, validated tool about life insurance and genetic counseling.

## 5 CONCLUSION

In this study, patient phenotype was the primary driver of whether cardiovascular genetic counselors discussed life insurance implications with their patients, having a larger impact than patient age or nationality of the genetic counselor. Our findings may support genetic counselors and similar providers in prioritizing their time spent on these discussions.

## AUTHOR CONTRIBUTIONS

Conceptualization: SAC, SJL, MM. Survey Construction and Implementation: SAC, SJL, MM, SC, GW. Data Curation: SAC, MM. Data Analysis and Validation: SAC, MM, SC. Writing: SAC, SJL, MM, SC, GW.

## FUNDING INFORMATION

No funding was secured for this study.

## CONFLICT OF INTEREST STATEMENT

Dr. Webster received support from Solid Biosciences, Inc. for consulting work unrelated to this study.

## ETHICS STATEMENT

This study was reviewed by the Ann and Robert H. Lurie Children's Hospital Institutional Review Board (#2024‐6785) and was designated as exempt.

## Supporting information


Appendix S1

## Data Availability

The data that support the findings of this study are available on request from the corresponding author.
