# Supplementary material for: Cardiovascular genetic counselor decision making about discussing life insurance with patients
Source: J Genet Couns. 2025 Nov 25;34(6):e70146. doi: 10.1002/jgc4.70146 (PMC12645423; doi:10.1002/jgc4.70146)
Supplement: Supplementary file 1 — Appendix S1 [file JGC4-34-0-s001.docx]

**SUPPLEMENTAL MATERIAL**

Cardiovascular genetic counselor decision-making

about discussing life insurance with patients

Sara Cherny, Sarah Jurgensmeyer Langas, Miguel Moran, Susan Christian, Gregory Webster

**Contents**

[Supplemental Table 1: Survey Instrument 2](#_Toc201075347)

[Supplemental Table 2: Likert Scale Dichotomization 5](#_Toc201075348)

[Supplemental Table 3: Sensitivity Analysis for Likert Scale Dichotomization 6](#_Toc201075349)

[Supplemental Table 4: Participant Location 7](#_Toc201075350)

# Supplemental Table 1: Survey Instrument

| **Question Number** | **Question** | **Response Choices** |
| --- | --- | --- |
| 1 | What year did you graduate from a genetic counseling graduate program? | Free text |
| 2 | In what country do you practice? | Australia  Canada  United Kingdom (England, Ireland, Northern Ireland, Scotland)  USA  Other |
| 3 | If Other, enter here | Free text |
| 4 | If you practice in the US, in what state/territory do you live? | {Standard U.S. choice list} |
| 5 | In what setting do you primarily practice? | Academic center  Private Practice  Commercial Lab  Other |
| 6 | If Other, enter here | Free text |
| 7 | What type of non-syndromic cardiac patients have you seen within the last year? Check all that apply. | Aortopathy/connective tissue  Arrhythmia  Cardiomyopathy  Congenital Heart Disease  Familial Hypercholesterolemia/dyslipidemia |
| 8 | What age of patients do you PREDOMINANTLY see? | Pediatric (0-17 years)  Adult (18+ years) |
|  | | |
| For the following questions, please consider potential impacts on life insurance such as an inability to obtain a policy or being able to obtain a policy but only at a higher premium. | | |
| *I discuss potential life insurance implications of genetic testing in the following contexts:* | | |
| 9 | Genetic testing in phenotype positive children (e.g. a 5yo with phenotype positive Long QT Syndrome) | Likert: Never… always* |
| 10 | Genetic testing in phenotype positive adults (e.g a 35yo with phenotype positive Long QT Syndrome) | Likert: Never… always* |
| 11 | Genetic testing in phenotype  negative children (e.g a 5yo with  a KCNQ1 pathogenic variant and  no phenotype) | Likert: Never… always* |
| 12 | Genetic testing in phenotype  negative adults (e.g. a 35yo with  a KCNQ1 pathogenic variant and  no phenotype) | Likert: Never… always* |
| *I discuss potential life insurance implications of genetic testing in the following contexts:* | | |
| 13 | Family variant testing | Likert: Never… always* |
| 14 | Panel testing | Likert: Never… always* |
| 15 | Exome/genome testing | Likert: Never… always* |
|  | | |
| The following are reasons why a genetic counselor might NOT discuss life insurance with  patients. Which of the following apply to you? | | |
| 16 | Not enough time | Likert: Don’t agree at all…very much agree* |
| 17 | Sometimes I forget | Likert: Don’t agree at all…very much agree* |
| 18 | Discussing life insurance might deter the patient from having genetic testing | Likert: Don’t agree at all…very much agree* |
| 19 | I do not feel confident in my  knowledge of life insurance | Likert: Don’t agree at all…very much agree* |
| 20 | The laws change too quickly | Likert: Don’t agree at all…very much agree* |
| 21 | The life insurance system is too  complicated | Likert: Don’t agree at all…very much agree* |
| 22 | The patient phenotype is already  a reason to affect their ability to  get life insurance | Likert: Don’t agree at all…very much agree* |
|  | | |
| 23 | I have had one or more patients decline genetic testing due to fear of life insurance discrimination. | Yes  No  Not sure |
| 24 | I have had one or more patients who were unable to Yes  obtain life insurance due to genetic test results. | Yes  No  Not sure |
| 25 | Does your team/department/employer have an established policy regarding communication with patients about life insurance implications of genetic testing? | Yes  No  Not sure |
|  | | |
| How strongly do you agree with the following questions? | | |
| 26 | My genetic counseling training included education about genetic non-discrimination laws | Likert: Don’t agree at all…very much agree* |
| 27 | My genetic counseling training included education about potential life insurance  implications of genetic testing | Likert: Don’t agree at all…very much agree* |
| 28 | I know where to find information about genetic non-discrimination laws in my country | Likert: Don’t agree at all…very much agree* |
| 29 | I know where to find information about genetic non-discrimination laws in my state | Likert: Don’t agree at all…very much agree* |
|  | | |
| 30 | Who should be MOST responsible for providing education and information about genetics and life insurance to genetic counselors? | Genetic counseling graduate programs  Genetic counseling education regulators such as Accreditation Council for Genetic Counseling (ACGC) and American Board of Genetic Counseling(ABGC)  Professional genetic counseling societies such as National Society Genetic Counselors (NSGC) and Canadian Association of Genetic Counsellors (CAGC)  Employing institution  Department leadership  Life insurance companies  Other |
| 31 | If Other, enter here |  |
| 32 | Does your country allow use of or access to genetic testing results in determining life insurance eligibility or cost? | Yes  No  Not sure |
| 33 | If you are counseling in the US, does your state allow life insurance companies to use genetic testing results in determining life insurance eligibility or cost? | Yes  No  Not sure |
| 34 | If you practice in multiple states, does your counseling regarding life insurance eligibility or cost change depending on the patient state of  residence? | Yes  No  Not sure |
|  | | |
| 35 | Which best describes your own life insurance status? | Through employer  Obtained independently  Insured through spouse  Insured through other family member  Do not have life insurance  More than one of the above |
| 36 | Which best describes your gender identity? | Agender  Genderfluid  Man  Non-binary  Pangender  Queer  Transgender  Two spirit  Woman  Something we have not listed  Prefer not to answer |
| 37 | How would you describe your ancestry? | Black, African American, or African descent  East Asian  European  Hispanic or Latine  Jewish  Middle Eastern or North African  Native Hawaiian or Pacific Islander  South Asian or Indian  West Asian  Something we have not listed here  Prefer not to answer |
| 38 | Thank you for participating in this survey! If you  have comments please enter them here. | Free text |

* For full Likert scales, see Supplemental Table 2.

# Supplemental Table 2: Likert Scale Dichotomization

| “Always… never” scale | In manuscript | Sensitivity Analysis #1 | Sensitivity Analysis #2 |
| --- | --- | --- | --- |
| Always | 1 | 1 | 1 |
| Often | 1 | 1 | 1 |
| Sometimes | 0 | 1 | Excluded |
| Rarely | 0 | 0 | 0 |
| Never | 0 | 0 | 0 |
| N/A | Excluded | Excluded | Excluded |
|  |  |  |  |
| “Agree… disagree” scale | **In manuscript** | **Sensitivity Analysis #1** | **Sensitivity Analysis #2** |
| Very Much Agree | 1 | 1 | 1 |
| Somewhat Agree | 1 | 1 | 1 |
| Neither Agree nor Disagree | 0 | 1 | Excluded |
| Somewhat Disagree | 0 | 0 | 0 |
| Disagree | 0 | 0 | 0 |
| N/A | Excluded | Excluded | Excluded |

The second column is the 5-point Likert dichotomization used for quantitative analysis in the manuscript. The third and fourth columns were used for sensitivity analysis (see Supplemental Tables 3A and 3B).

# Supplemental Table 3A: Sensitivity Analysis for Likert Scale Dichotomization (“Always… never” scale)

|  | Manuscript dichotomization | | Sensitivity analysis #1 | | Sensitivity analysis #2 | |
| --- | --- | --- | --- | --- | --- | --- |
| **Patient Phenotype Effect on LI Discussion: Adult and Pediatric GCs** | | | | | | |
| Adult Positive | 46% vs. 17% | p = 0.008 | 68% vs. 38% | p = 0.007 | 46% vs. 17% | p = 0.003 |
| Pediatric Positive | 44% vs. 12% | p = 0.003 | 61% vs. 38% | p = 0.047 | 44% vs. 13% | p = 0.004 |
| Adult Negative | 79% vs. 75% | p = 0.682 | 90% vs 96% | p = 0.369 | 79% vs. 75% | p = 0.436 |
| Pediatric Negative | 78% vs. 73% | p = 0.628 | 85% vs. 96% | p = 0.127 | 77% vs. 73% | p = 0.194 |
|  | | | | | | |
| **Patient Phenotype Effect on LI Discussion: US and non-US GCs** | | | | | | |
| Adult Positive | 33% vs. 61% | p = 0.005 | 55% vs. 81% | p = 0.011 | 33% vs. 61% | p = 0.004 |
| Pediatric Positive | 32% vs. 46% | p = 0.195 | 51% vs. 65% | p = 0.219 | 32% vs. 46% | p = 0.167 |
| Adult Negative | 77% vs. 81% | p = 0.685 | 90% vs. 94% | p = 0.592 | 77% vs. 81% | p = 0.586 |
| Pediatric Negative | 79% vs. 70% | p = 0.375 | 92% vs. 78% | p = 0.063 | 79% vs. 70% | p = 0.08 |
|  | | | | | | |
| **Test Type Effect on LI Discussion: Adult and Pediatric GCs** | | | | | | |
| Family Variant | 92% vs. 85% | p = 0.216 | 98% vs. 92% | p = 0.162 | 98% vs. 91% | p = 0.152 |
| Panel | 62% vs. 31% | p = 0.005 | 84% vs. 73% | p = 0.230 | 71% vs. 53% | p = 0.039 |
| Exome/Genome | 72% vs. 60% | p = 0.248 | 87% vs. 80% | p = 0.403 | 85% vs. 75% | p = 0.324 |
|  | | | | | | |
| **Test Type Effect on LI Discussion: US and non-US GCs** | | | | | | |
| Family Variant | 91% vs. 91% | p = 0.962 | 97% vs. 97% | p = 0.939 | 96% vs. 97% | p = 0.943 |
| Panel | 51% vs. 63% | p = 0.298 | 80% vs. 84% | p = 0.589 | 72% vs. 80% | p = 0.448 |
| Exome/Genome | 70% vs. 68% | p = 0.885 | 87% vs. 80% | p = 0.856 | 83% vs. 81% | p = 0.853 |

# Supplemental Table 3B: Sensitivity Analysis for Likert Scale Dichotomization (“Agree… disagree” scale)

|  | Manuscript dichotomization | | Sensitivity analysis #1 | | Sensitivity analysis #2 | |
| --- | --- | --- | --- | --- | --- | --- |
| **Reasons a Genetic Counselor Might Not Discuss Life Insurance: Adult and Pediatric GCs** | | | | | | |
| Time | 18% vs. 35% | p. = 0.691 | 37% vs. 50% | p = 0.224 | 33% vs. 41% | p = 0.461 |
| Forget | 47% vs. 58% | p = 0.351 | 53% vs. 69% | p = 0.131 | 50% vs. 65% | p = 0.192 |
| Deter patient from test | 5% vs. 8% | p = 0.638 | 11% vs. 23% | p = 0.094 | 6% vs. 9% | p = 0.539 |
| Confidence | 20% vs. 27% | p = 0.446 | 33% vs. 35% | p = 0.849 | 23% vs. 29% | p = 0.528 |
| Laws change quickly | 9% vs. 12% | p = 0.768 | 22% vs. 35% | p = 0.201 | 11% vs. 15% | p = 0.616 |
| Complicated system | 20% vs 27% | p = 0.446 | 36% vs. 38% | p = 0.802 | 24% vs. 30% | p = 0.515 |
| Patient phenotype | 88% vs. 96% | p = 0.288 | 93% vs. 100% | p = 0.186 | 93% vs. 100% | p = 0.185 |
|  | | | | | | |
| **Reasons a Genetic Counselor Might Not Discuss Life Insurance: US and non-US GCs** | | | | | | |
| Time | 28% vs 41% | p = 0.190 | 39% vs. 41% | p = 0.897 | 32% vs. 41% | p = 0.366 |
| Forget | 49% vs. 50% | p = 0.957 | 55% vs. 59% | p = 0.673 | 52% vs. 55% | p = 0.795 |
| Deter patient from test | 7% vs. 3% | p = 0.452 | 16% vs. 6% | p = 0.175 | 7% vs. 3% | p = 0.413 |
| Confidence | 19% vs. 28% | p = 0.286 | 29% vs. 44% | p = 0.134 | 21% vs. 33% | p = 0.206 |
| Laws change quickly | 8% vs. 16% | p = 0.215 | 21% vs. 34% | p = 0.153 | 9% vs. 19% | p = 0.171 |
| Complicated system | 20% vs. 25% | p = 0.573 | 35% vs. 41% | p = 0.559 | 24% vs. 30% | p = 0.541 |
| Patient phenotype | 93% vs 81% | p = 0.028 | 97% vs. 88% | p = 0.023 | 98% vs. 87% | p = 0.020 |

Divisions for the column headers are provided in Supplemental Table 2. P-values < 0.05 in **blue**.

# Supplemental Table 4: Participant Location

Participants were asked to self-identify their country of practice. For US-based participants, they were asked to provide the state of practice as well.

| Arizona | 1 |
| --- | --- |
| California | 18 |
| Colorado | 1 |
| Connecticut | 1 |
| Georgia | 2 |
| Illinois | 10 |
| Indiana | 3 |
| Iowa | 1 |
| Kentucky | 1 |
| Maryland | 3 |
| Massachusetts | 3 |
| Michigan | 3 |
| Minnesota | 3 |
| Missouri | 1 |
| Nebraska | 1 |
| New York | 3 |
| North Carolina | 2 |
| Ohio | 10 |
| Oregon | 1 |
| Pennsylvania | 4 |
| South Carolina | 2 |
| Texas | 2 |
| Utah | 2 |
| Virginia | 3 |
| Wisconsin | 6 |
| Not completed | 2 |
| Total | 89 |
